# A Small Molecule-Screening Pipeline to Evaluate the Therapeutic Potential of 2-Aminoimidazole Molecules Against *Clostridium difficile*

**DOI:** 10.3389/fmicb.2018.01206

**Published:** 2018-06-06

**Authors:** Rajani Thanissery, Daina Zeng, Raul G. Doyle, Casey M. Theriot

**Affiliations:** ^1^Department of Population Health and Pathobiology, College of Veterinary Medicine, North Carolina State University, Raleigh, NC, United States; ^2^Agile Sciences, Inc., Raleigh, NC, United States

**Keywords:** *C. difficile*, small molecules, 2-aminoimidazole, growth, toxin, sporulation

## Abstract

Antibiotics are considered to be the first line of treatment for mild to moderately severe *Clostridium difficile* infection (CDI) in humans. However, antibiotics are also risk factors for CDI as they decrease colonization resistance against *C. difficile* by altering the gut microbiota and metabolome. Finding compounds that selectively inhibit different stages of the *C. difficile* life cycle, while sparing the indigenous gut microbiota is important for the development of alternatives to standard antibiotic treatment. 2-aminoimidazole (2-AI) molecules are known to disrupt bacterial protection mechanisms in antibiotic resistant bacteria such as *Pseudomonas aeruginosa, Acinetobacter baumannii*, and *Staphylococcus aureus*, but are yet to be evaluated against *C. difficile*. A comprehensive small molecule-screening pipeline was developed to investigate how novel small molecules affect different stages of the *C. difficile* life cycle (growth, toxin, and sporulation) *in vitro*, and a library of commensal bacteria that are associated with colonization resistance against *C. difficile*. The initial screening tested the efficacy of eleven 2-AI molecules (compound 1 through 11) against *C. difficile* R20291 compared to a vancomycin (2 μg/ml) control. Molecules were selected for their ability to inhibit *C. difficile* growth, toxin activity, and sporulation. Further testing included growth inhibition of other *C. difficile* strains (CD196, M68, CF5, 630, BI9, M120) belonging to distinct PCR ribotypes, and a commensal panel (*Bacteroides fragilis, B. thetaiotaomicron, C. scindens, C. hylemonae, Lactobacillus acidophilus, L. gasseri, Escherichia coli, B. longum* subsp. *infantis*). Three molecules compound 1 and 2, and 3 were microbicidal, whereas compounds 4, 7, 9, and 11 inhibited toxin activity without affecting the growth of *C. difficile* strains and the commensal microbiota. The antimicrobial and anti-toxin effects of 2-AI molecules need to be further characterized for mode of action and validated in a mouse model of CDI.

## Introduction

*Clostridium difficile* is the leading cause of nosocomial and antibiotic associated infectious diarrhea worldwide. *C. difficile* causes over 450,000 infections and 29,000 deaths annually in the United States (Lessa et al., 2015; McDonald et al., 2018). The incidence, severity, and recurrence rates have increased markedly with the emergence of epidemic strains, and exposure to classic risk factors such as recent antibiotic use, advanced age, and prior hospitalization (Stabler et al., 2006; Ananthakrishnan, 2011; Loo et al., 2011). In addition, *C. difficile* is now increasingly being linked to community acquired cases of colitis in individuals not exposed to typical risk factors (CDC, 2008; Gupta and Khanna, 2014; Knetsch et al., 2017; McDonald et al., 2018). The changing epidemiology, and the subsequent challenges in the treatment of this infection has prompted the Centers for Disease Control and Prevention (CDC) to classify *C. difficile* as an urgent threat to public health (CDC, 2013).

*Clostridium difficile* infection (CDI) is initiated by spores that are highly resistant to various physical and chemical stressors, enabling them to persist in the environment, and play a key role in disease transmission (Baines et al., 2009; Loo et al., 2011; Deakin et al., 2012; Paredes-Sabja et al., 2014). In the gut, the presence of calcium, glycine, and primary bile acids such as taurocholate sensed by the germinant receptor CspC enables *C. difficile* spores to germinate into metabolically active vegetative cells (Sorg and Sonenshein, 2008; Francis et al., 2013; Kochan et al., 2017). However, the normal indigenous gut microbiota provides colonization resistance against *C. difficile* (Theriot et al., 2014; Buffie et al., 2015). Antibiotic mediated disruption of the gut microbiota and metabolome leads to a loss of colonization resistance favoring vegetative cell proliferation, and production of toxins that ultimately mediate disease (Antunes et al., 2011; Theriot et al., 2016). During CDI, *C. difficile* initiates the sporulation pathway forming metabolically dormant spores there by completing the life cycle. The signals that trigger the onset of sporulation are not well understood, however, substantial evidence supports the link between nutrient limitation or other stress factors with sporulation and virulence (Paredes-Sabja et al., 2014; Nawrocki et al., 2016). Current line of treatment for patients with CDI includes the antibiotics vancomycin, metronidazole, or fidaxomicin, which in approximately 20–30% of the patients is ineffective resulting in recurrence (Cohen et al., 2010; Lessa et al., 2015). The intrinsic damage caused by the current line of antibiotics on the gut microbiota, and its failure to restore colonization resistance is the major limiting factor in the treatment and management of CDI (DuPont, 2011). There are occasional reports of *C. difficile* having high MIC *in vitro* to the drugs used for its treatment (Baines et al., 2008; Martin et al., 2008; Snydman et al., 2012), however, to date treatment failures have not been linked to antimicrobial resistance. Considering the ease with which *C. difficile* spread globally in a short time span (He et al., 2013), coupled with the fact that antibiotics are risk factors, there is growing consensus for drug targets that selectively inhibit *C. difficile* vegetative cells and or virulence factors, while sparing the indigenous gut microbiota. Compounds that inhibit sporulation would also be beneficial as they would aid in the prevention of transmission and relapse.

Identifying potential drug targets against *C. difficile* is challenging because of the complex etiology, and the impact of risk factors that lead to the disease (Smits et al., 2016). Traditionally, MIC’s and kill assays were used in initial drug screening pipelines, which focuses only on the growth stage of the *C. difficile* life cycle. Here we present a comprehensive small molecule pipeline, which evaluates the activity of test compounds on three different stages of the *C. difficile* life cycle (growth kinetics, toxin activity, and sporulation), and how they impact the growth of *C. difficile* strains from distinct PCR ribotypes. Additionally, the pipeline evaluates how these small molecules alter the growth of other gut commensals that are associated with colonization resistance against *C. difficile*. The goal of the *in vitro* screening strategy described here is to screen and select promising compounds that are able to inhibit one or all of the steps in the *C. difficile* life cycle. Future work defining the mechanism of action of each compound and validating them in a mouse model of CDI is down stream of this pipeline.

2-aminoimidazole (2-AI) molecules have a unique mechanism of action by targeting two-component systems (TCSs), which are signaling pathways that allow bacteria to respond to environmental signals (antibiotics or quorum sensing molecules) there by inhibiting virulence responses such as antibiotic resistance, toxin secretion, and biofilm formation (Thompson et al., 2012). These processes are important in pathogenesis and survival of the pathogen within the host (Stock et al., 2000; Stephenson and Hoch, 2002; Beier and Gross, 2006). 2-AI molecules have been successfully used for antibiotic potentiation and anti-virulence activities against other antibiotic resistant bacteria such as *Pseudomonas aeruginosa, Acinetobacter baumannii*, and *Staphylococcus aureus*, but are yet to be evaluated against *C. difficile* (Rogers et al., 2010; Brackett et al., 2014; Draughn et al., 2017). *C. difficile* relies on TCS signaling pathways for toxin production that mediate disease, and sporulation which plays a key role in transmission and recurrence (Underwood et al., 2009; Darkoh et al., 2015, 2016). Therefore, we hypothesized that 2-AI molecules would be able to inhibit different stages of *C. difficile* life cycle namely toxin activity and sporulation. In this study, we started with eleven 2-AI molecules (compound 1 through 11) in our comprehensive screening pipeline, and tested their ability to inhibit *C. difficile* growth, toxin activity, and sporulation. Molecules that showed potent activity against *C. difficile* R20291 were further tested against other *C. difficile* strains (CD196, CF5, M68, BI9, 630, and M120) belonging to distinct PCR ribotypes, and an eight-member commensal library of bacteria associated with colonization resistance against *C. difficile*. Compound 1, 2, and 3 were found to inhibit growth kinetics, whereas compounds 4, 7, 9, and 11 inhibited toxin activity without affecting the growth of both *C. difficile* strains and commensals. Next steps include evaluation of each compound for the mechanism of action, and validation in a mouse model of CDI.

## Materials and Methods

### Bacterial Strains

#### *Clostridium difficile* Strains and Growth Conditions

*Clostridium difficile* strains selected from a range of PCR ribotypes, including epidemic (R20291 and M68), non-epidemic (CD196, CF5, and 630), current (R20291, M68, and BI9), and a genetically divergent strain (M120) were used in these studies. R20291, CD196, CF5, M68, 630, BI-9, and M120 belongs to ribotypes 027, 027, 017, 017, 012, 001, and 078, respectively. The origin and reference details of the isolates can be obtained from **Table 2** of our previous publication (Sebaihia et al., 2006; Stabler et al., 2009; He et al., 2010; Thanissery et al., 2017). All assays using *C. difficile* were started from spore stocks. Spores were prepared and tested for purity as described previously (Perez et al., 2011; Thanissery et al., 2017). Briefly, individual *C. difficile* strains were grown anaerobically in 2 ml Columbia broth at 37°C for 12 h and further sub-cultured into 40 ml Clospore media in which it was allowed to sporulate for 5–7 days. Spores were harvested by centrifugation and subjected to 3–5 washes with sterile cold water. Spore stocks were stored at 4°C in sterile water until use. The spores were heat treated (65°C for 20 min) to kill vegetative cells, before enumeration and testing for purity. The viable spores were enumerated on brain heart infusion (BHI, Becton, Dickinson and Company, Sparks, MD, United States) media supplemented with 100 mg/L L-cysteine and 0.1% taurocholate. To ensure purity, spores were plated on BHI media plus 100 mg/L L-cysteine, with and without spore germinant (0.1% taurocholate). The purified spores were further examined under phase contrast microscope in which non-germinated intact spores appeared as phase bright bodies. *C. difficile* cultures for the assays were prepared by inoculating spores on BHI media supplemented with 100 mg/L L-cysteine and 0.1% taurocholate. The plates were incubated anaerobically overnight at 37°C, and isolated colonies from these plates were used to prepare *C. difficile* inoculum in BHI broth with 100 mg/L L-cysteine.

#### Commensal Library Strains and Growth Conditions

Eight different non-*C. difficile* strains that are members of the healthy human gut microbiota belonging to four dominant bacterial phyla including Bacteroidetes, Firmicutes, Proteobacteria, and Actinobacteria were used to determine MIC’s of various 2-AI molecules. Strain details and sources are shown in **Table 1**. *Bacteroides fragilis* NCTC 9343, and *Bacteroides thetaiotaomicron* VPI-5482 were obtained from Eric Martens (University of Michigan, United States). *Clostridium hylemonae* TN-271 was obtained from Joson M. Ridlon (University of Illinois Urbana-Champaign, United States). *Lactobacillus acidophilus* ATCC 700396, *Lactobacillus gasseri* ATCC 33323, and *Bifidobacterium longum* subsp. *infantis* DSM 20090 were obtained from Rodolphe Barrangou (North Carolina State University, United States). *Clostridium scindens* (ATCC 35704, Cat # 35704) and *Escherichia coli* (Cat # BAA 2649) were purchased from American Type Culture Collection. All strains were maintained as 15% glycerol stock in -80°C until use. Working stocks of *Bacteroides species* were prepared in tryptone-yeast extract- glucose (TYG) media (Martens et al., 2008). *C. scindens, C. hylemonae*, and *E. coli* were grown in BHI plus 100 mg/L L-cysteine (Barefoot and Klaenhammer, 1983; Ridlon et al., 2010). *Lactobacillus acidophilus*, and *L. gasseri* were grown in de Man, Rogosa, and Sharpe broth (MRS, Becton, Dickinson and Company, Sparks, MD, United States), (Barefoot and Klaenhammer, 1983). *Bifidobacterium longum* subsp. *infantis* were grown in MRS supplemented with 500 mg/L L-cysteine (Ventura et al., 2003).

**Table 1:** Commensal microbiota library.

Phyla	Bacteria	Strain^∗^	Description	Nucleotide accession no.
				(complete genome)/Reference
Bacteroidetes	*Bacteroides fragilis*	NCTC 9343	Type strain, appendix abscess	GenBank, CR626927
Bacteroidetes	*Bacteroides thetaiotaomicron*	VPI-5482	Type strain, human feces	Xu et al., 2003
Firmicutes	*Lactobacillus acidophilus*	ATCC 700396/NCFM	Infant feces	Altermann et al., 2005
Firmicutes	*Lactobacillus gasseri*	ATCC 33323	Type strain	GenBank, CP000413
Firmicutes	*Clostridium scindens*	ATCC 35704	Type strain, human feces	GenBank, ABFY02000000
Firmicutes	*Clostridium hylemonae*	TN-271	Type strain, human feces	GenBank, AB023972^∗∗^
Proteobacteria	*Escherichia coli*	ATCC BAA 2649	Not type strain	
Actinobacteria	*Bifidobacterium longum* subsp. *infantis*	DSM 20090	Intestine of infants	Mattarelli et al., 2008

*^∗^ATCC, American Type Culture Collection; NCTC, National Collection of Type Cultures; DSM, Leibniz-Institute DSMZ-German Collection of Microorganisms and Cell Cultures.*

*^∗∗^16S rRNA, partial sequence.*

### Small Molecule Preparation

**FIGURE 1:**
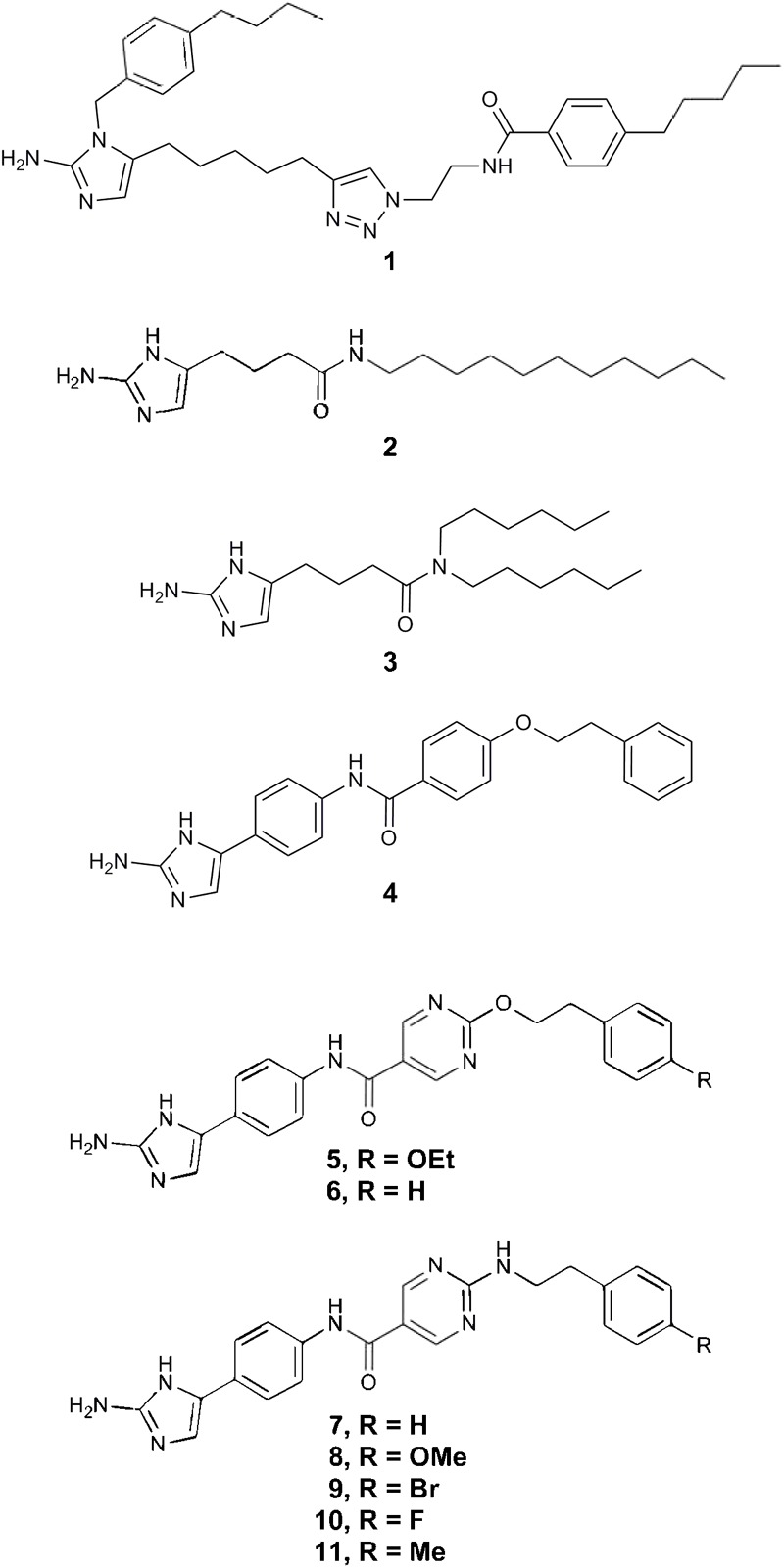
2-aminoimidazole chemical structures. The chemical structures of all eleven 2-AI molecules are illustrated in this figure.

2-AI molecules compound 1, 2, 3, 4, 5, 6, 7, 8, 9, 10, and 11 (A kind gift of Agile Sciences Inc., Raleigh, NC, United States, **Figure 1**) were provided as a 400 μg/mL stock in 10% dimethyl sulfoxide (DMSO, Sigma-Aldrich Co., St. Louis, MO, United States) and were stored at -20°C until use. For all assays, the test compounds were used at a maximum final concentration of 10 μg/mL to ensure efficacy when compared to vancomycin that is currently used for the treatment of CDI (Cohen et al., 2010). Vancomycin (Sigma-Aldrich, St. Louis, MO, United States) was used as a positive control in all assays. Stock solution of vancomycin (8 mg/mL) was diluted in ultrapure water, filter sterilized and stored at 4°C for a week.

### Microbroth Dilution for Minimum Inhibitory Concentration Assay

Minimum inhibitory concentration was determined using a modified Clinical and Laboratory Standard Institute (CLSI) broth microdilution method. Test medium used for all *Clostridia* were BHI with 100 mg/L L-cysteine. Bacteroides were grown in Yeast extract casitone fatty acid medium. *Lactobacillus* sp. were grown in MRS. The same medium was supplemented with 500 mg/L L-cysteine for growing *B. infantis.* The inoculum was prepared by the direct colony suspension method. All cell concentrations were adjusted to ∼5 × 10^5^ CFU/mL. An anaerobic environment was maintained at all times using an anaerobic chamber (Coy Industries). An incubation temperature of 37°C was used for all strains. The plates were prepared fresh by making 2-AI molecules or vancomycin dilution stocks in the test media, and adding 90 μL to each well such that the final concentration of the test compounds after the addition of cells (10 μL) ranged from 0.08 to 10 μg/mL. Positive controls included inoculated cells only (in test media to check for media adequacy), and solvent (0.25% DMSO). Uninoculated test media for each strain was used as a negative control to check for sterility. The assay plates were then sealed using a sterile polyester film (VWR, cat # 89134-432) before placing the lid to prevent the panel from dehydrating during incubation. *C. difficile, B. fragilis, B. thetaiotaomicron, L. acidophilus, L. gasseri*, and *E. coli* were incubated for 24 h, whereas *C. scindens*, and *C. hylemonae*, were allowed to grow for 48 h. MICs were defined as the lowest concentration at which there was no visible growth. The end point optical density at 600 nm (OD_600_) of the plates was additionally recorded to measure turbidity.

### Growth Kinetics Inhibition Assay

The growth inhibition studies of *C. difficile* were done in a 96-well microtiter plate using previously published methods (Thanissery et al., 2017). All *C. difficile* strains were cultured overnight at 37°C in pre-reduced BHI plus 100 mg/L L-cysteine broth in an anaerobic chamber. Overnight *C. difficile* cultures were sub-cultured 1:10 into same media, and allowed to grow for 3 h anaerobically at 37°C. The culture was then diluted in fresh BHI so that the starting OD_600_ was 0.01. The cell suspension was added in triplicate to a 96-well plate at a final volume of 0.2 ml with the addition of test compound (final concentration: 10 μg/mL), solvent (0.25% DMSO) or vancomycin (final concentration: 2 μg/mL). Each plate contained control wells (without test compounds) and blank wells (without cells). The plates were sealed to ensure anaerobic conditions and passed outside the chamber to measure optical density 600 nm (OD_600_). The optical density was monitored every 30 min for 10 h, shaking the plate for 90 s before each reading, in a Tecan plate reader. A test plate containing 2-AI or vancomycin in media was run before the assay to measure the optical density and ensure the stability of the compounds over the incubation period. After 24 h, the plates were removed from the plate reader and stored in -80°C until use for measuring toxin activity from the culture supernatants.

### Toxin Activity Inhibition Assay

Toxin activity was measured by a Vero cell cytotoxicity assay (Winston et al., 2016; Thanissery et al., 2017). Vero cells were grown and maintained in DMEM media (Gibco Laboratories, 11965-092) with 10% fetal bovine serum (Gibco Laboratories, 16140-071) and 1% Penicillin streptomycin solution (Gibco Laboratories, 15070-063). Cells were incubated with 0.25% trypsin (Gibco Laboratories, 25200-056), washed with 1X DMEM media, and harvested by centrifugation 1,000 RPM for 5 min. Cells were plated at 1 × 10^4^ cells per well in a 96-well flat bottom microtiter plate (Corning, 3596) and incubated overnight at 37°C/5% CO_2_. Growth inhibition kinetics assay plates were defrosted on ice and then centrifuged at 1,750 RPM for 5 min to pellet vegetative *C. difficile*. Culture supernatants were collected from each well and serially diluted by 10-fold to a maximum of 10^-6^ using 1X PBS. Sample dilutions were incubated 1:1 with PBS (for all dilutions) or antitoxin (performed for 10^-1^ and 10^-4^ dilutions only, TechLabs, T5000) for 40 min at room temperature. Following incubation, these admixtures were added to the Vero cells. After an overnight incubation at 37°C/5% CO_2_, plates were viewed under 200× magnification for Vero cell rounding. The cytotoxic titer was defined as the reciprocal of the highest dilution that produced rounding in 80% of Vero cells for each sample. Vero cells treated with purified *C. difficile* toxins (A and B) and antitoxin (List Biological Labs, 152C and 155C; TechLabs, T5000) were used as controls. A test cytotoxicity assay was run prior to assays to ensure that the 2-AI molecules did not affect the cytoskeleton of Vero cells at the tested concentrations.

### Kill Kinetics Assay

#### Measurement of OD_600_ Using Plate Reader

Kill kinetics of *C. difficile* were analyzed on a 96-well plate using a modified growth inhibition assay protocol. Briefly, overnight *C. difficile* cultures were back-diluted 1:25 into pre-reduced BHI plus 100 mg/L L-cysteine broth and allowed to grow until it reaches mid log (OD_600_ of 0.45–0.50). The cells were added in triplicates to a 96-well plate at the same volume and concentrations of test compound, solvent, or vancomycin as used in the growth kinetics inhibition assay. Each plate also contained control wells (without test compounds) and blank wells (without cells). The optical density was monitored every 30 min for 12 h, shaking the plate for 90 s before each reading, in a Tecan plate reader.

#### *C. difficile* Bacterial Enumeration

Plates were prepared as described here previously for measurement of OD_600_ using a plate reader. Six hours later, 25 μL aliquots were removed from each treatment, serially diluted 10-fold in phosphate buffered saline (PBS), and plated on BHI plus 100 mg/L L-cysteine and 0.1% taurocholate using a track dilution method (Jett et al., 1997). This method involved plating 10 μL of six dilutions on separate tracks of a single square plate (Genesee Scientific, Cat # 26-275). The dilution plate was then heat treated at 65°C for 20 min to kill all vegetative cells. Following heat treatment, the cells were plated on BHI plus 100 mg/L L-cysteine and 0.1% taurocholate. All plates were incubated at 37°C for 24 h anaerobically. Plates were counted the next day to enumerate total vegetative cells plus spores in the unheated samples, and total spores in the heat-treated samples.

### Sporulation Inhibition Assay

The sporulation assay is modified from a method previously described as spore inducing and quantification using heat resistance (Shen et al., 2016). Briefly, R20291 spores were streaked on BHI plates containing 100 mg/L L-cysteine plus 0.1% taurocholate and incubated anaerobically for 24 h. The colonies were sub-cultured into 2 mL BHI plus 100 mg/L L-cysteine and were allowed to grow for 4 to 5 h. The turbid culture was centrifuged for 5 min, and the pellet was resuspended in 70:30 broth [per liter contained 63 g Bacto Peptone, 3.5 g Protease Peptone, 0.7 g NH 4 SO 4, 1.6 g Tris Base, 11.1 g BHI Broth, 1.5 g Yeast Extract, supplemented with 3 mL 10% (w/v) Cysteine] to an OD_600_ of ∼0.5. Resuspended cultures (195 μL) with or without test compounds (final concentration: 10 μg/mL), vancomycin (2 μg/mL), or solvent (0.25% DMSO) were incubated at 37°C for 24 h anaerobically. The samples after incubation (20 μL) were serially diluted 10-fold, and 4 μL were plated on BHI plates containing 100 mg/L L-cysteine plus 0.1% taurocholate. The dilution plate was passed out of the chamber for heat treatment at 65°C for 20 min. Four μL from each dilution was plated on BHI plates containing 100 mg/L L-cysteine plus 0.1% taurocholate. All plates were incubated anaerobically at 37°C for 24 h. The number of colony forming units (CFUs) were counted on the lowest dilution in which colonies were visible to determine the CFU/mL of total vegetative cells and spores from the unheated samples and spores only from the heat-treated samples.

### Statistical Analysis

Statistical tests were performed using Prism version 7.0a for Mac OS X (GraphPad Software, La Jolla, CA, United States). Significance between treatments and solvent control for toxin activity assay (**Figure 3B**), bacterial enumeration for kill kinetics (**Figure 4**), and sporulation assay (**Figure 5**) were calculated by Student’s parametric *t*-test with Welch’s correction. Statistical significance was set at a *p*-value of <0.05 for all analyses (^∗^*p* < 0.05, ^∗∗^*p* < 0.01, ^∗∗∗^*p* < 0.001, ^∗∗∗∗^*p* < 0.0001). All assays were done in triplicate.

## Results

### Development of a Screening Pipeline to Test Small Molecule Activity Against Different Stages of the *C. difficile* Life Cycle *in Vitro*

**FIGURE 2:**
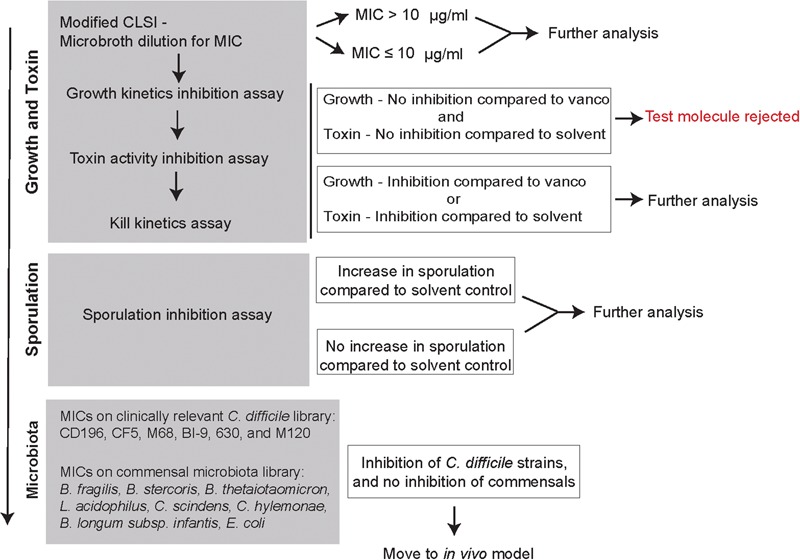
*In vitro* screening pipeline to evaluate 2-aminoimidazole molecules as potential non-antibiotic therapeutics for *C. difficile* infection. Flowchart is designed as an *in vitro* screening pipeline to evaluate small molecules against *C. difficile*. The gray boxes represent the different protocols used to determine how the small molecules affect different stages of *C. difficile* life cycle (growth, toxin, and sporulation) and other members of the gut microbiota. Based on the results, at each box a decision is made whether to advance molecules for further screening with the goal of identifying suitable molecules to be advanced to mouse model testing.

**Figure 2** is an overview of the small molecule-screening pipeline that was developed and implemented in this study. The gray boxes represent the different assays that were used to interrogate how small molecules were able to alter different stages of the *C. difficile* life cycle including growth, toxin, and sporulation. We also evaluated their activity against other *C. difficile* strains (CD196, CF5, M68, BI9, 630, M120), and commensals from the gut microbiota (*B. fragilis, B. thetaiotaomicron, L. acidophilus, L. gasseri, C. scindens, C. hylemonae, E. coli*, and *B. longum* subsp. *infantis*) (**Table 1**). All small molecules begin at the first step, screening on a 96-well plate to determine MICs using a microbroth dilution technique. A MIC of 10 μg/ml was considered an initial cut-off for activity when compared to the reference drug vancomycin, which is currently used to treat patients with CDI. This dose was selected because it was hard to sustain concentrations above 10 μg/ml in animal studies based on previous studies with structurally similar compounds. All molecules along with vancomycin (2 μg/ml) and the solvent (0.25% DMSO) were moved down the pipeline, and assayed for growth kinetics inhibition, toxin activity inhibition, and kill kinetics. Molecules that either inhibited growth and or toxin activity were advanced to the next step in the pipeline. A sporulation induction assay was used to determine if the small molecules were able to alter sporulation. All other molecules were moved to the next step in the pipeline where they were screened for activity against other clinical *C. difficile* strains, and a commensal microbiota library. Molecules that show promising antimicrobial or anti-toxin activity sparing the commensals in this pipeline will be further evaluated *in vivo* in a mouse model of CDI.

### 2-Aminoimidazole Molecules Alter *C. difficile* R20291 Growth and Toxin Activity

The MICs for eleven 2-AI molecules with *C. difficile* are shown in the **Table 2**. Compounds 1, 2, and 3 were the most active against *C. difficile* with MICs ranging from 2.5 to 5 μg/ml. *C. difficile* was not susceptible to all other 2-AI molecules. The control vancomycin had a MIC of 0.15–0.31 μg/ml, and the solvent control (0.25% DMSO) did not inhibit *C. difficile*.

**Table 2:** Minimum inhibitory concentration of 2-aminoimidazole molecules against *C. difficile* strain R20291 compared to vancomycin.

Test compound	MIC (μg/ml)
Vancomycin	0.15–0.31
1	2.5–5
2	5
3	5
4	>10
5	>10
6	>10
7	>10
8	>10
9	>10
10	>10
11	>10

*Minimum inhibitory concentration (MIC) was determined by broth microdilution as per modified CLSI guidelines for anaerobes. Data represent mean values from triplicate trials.*

**FIGURE 3:**
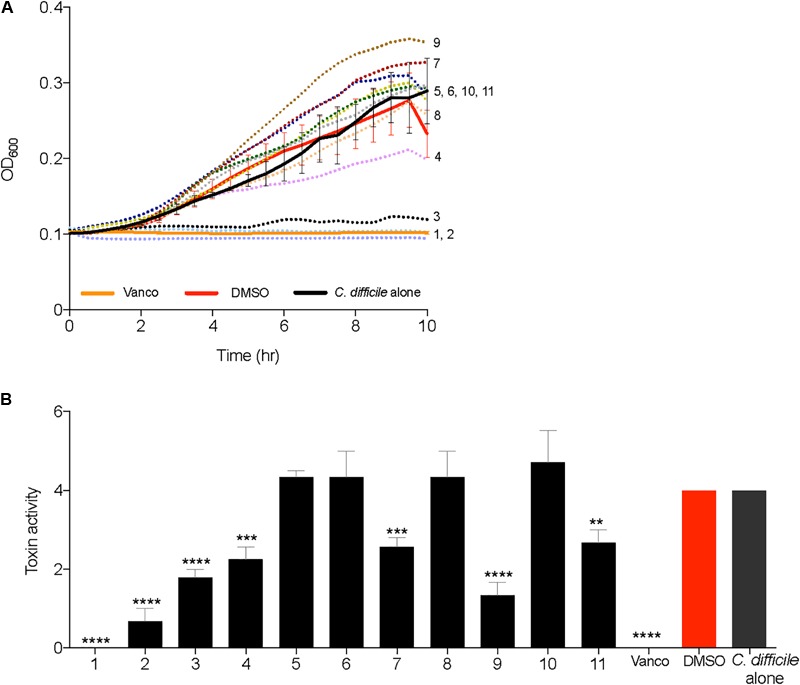
2-aminoimidazole molecules inhibit growth and toxin activity of *C. difficile*. **(A)** Inhibition of *C. difficile* R20291 growth (OD_600_) in BHI media supplemented with small molecules (Compound 1 through 11) at a concentration of 10 μg/ml, solvent 0.25% DMSO (DMSO), or 2 μg/ml Vancomycin (Vanco). **(B)** Culture supernatants after 24 h growth inhibition assays were used for Vero cell cytotoxicity assays to measure toxin activity. Toxin titer is expressed as log10 reciprocal dilution toxin per 100 μl of *C. difficile* culture supernatant. Data presented represents mean ± SEM of triplicate experiments. In **(B)** statistical significance between positive control (solvent) and treatment groups was determined by Student’s parametric *t-*test with Welch’s correction (^∗^*p* < 0.05, ^∗∗^*p* < 0.01, ^∗∗∗^*p* < 0.001, ^∗∗∗∗^*p* < 0.0001).

All 2-AI molecules were moved down the pipeline and tested in a *C. difficile* growth kinetics inhibition assay at a concentration of 10 μg/ml, along with vancomycin (2 μg/ml), and the solvent (0.25% DMSO). Supplementation of compounds 1, 2, and 3 inhibited the growth of *C. difficile* and was very similar to the vancomycin control (**Figure 3A**). There was no change in *C. difficile* growth kinetics in the presence of all other 2-AI molecules. Toxin activity was measured from culture supernatants of *C. difficile* supplemented with 2-AI molecules in **Figure 3A**. Diminished growth correlated with low toxin activity with the addition of compounds 1, 2, and 3 (**Figure 3B**). Interestingly, growth was unaffected by compounds 4, 7, 9, and 11 yet toxin activity was significantly reduced when compared to the solvent control. The addition of the solvent DMSO to media did not alter *C. difficile* growth or toxin activity. The cytotoxic activity was neutralized at all dilutions containing the sample and antitoxin confirming that the cell rounding was from *C. difficile* toxin. All molecules were advanced to the kill kinetics assay, the next step in the pipeline.

**FIGURE 4:**
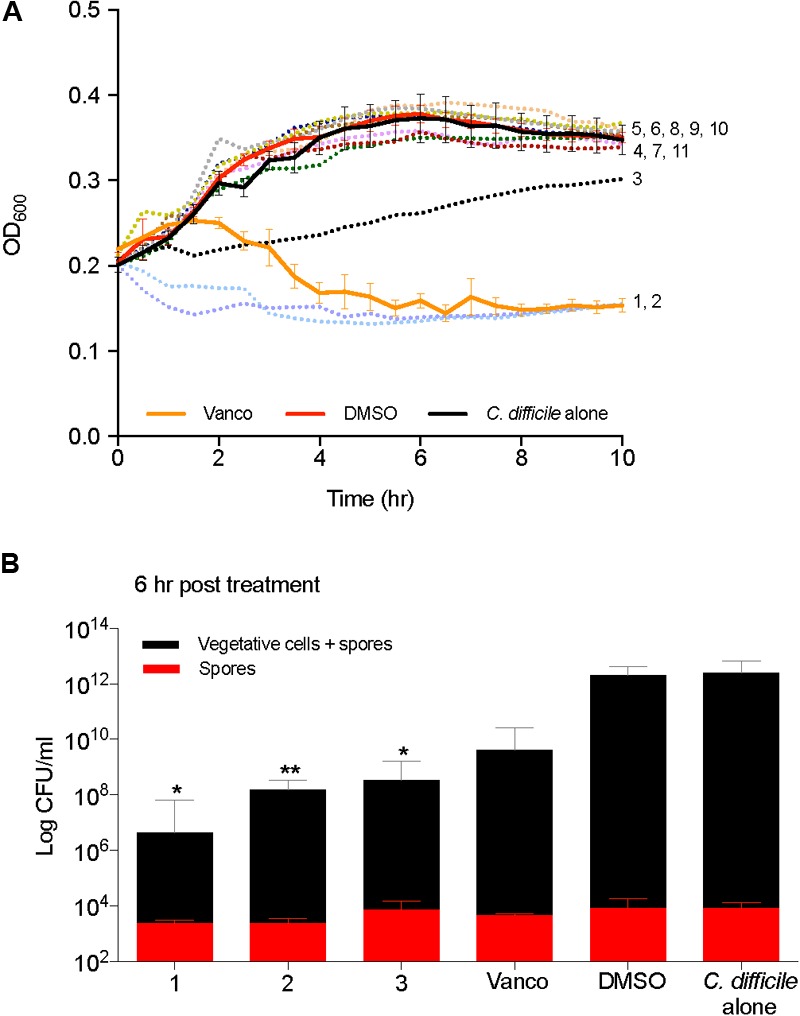
2-aminoimidazole molecules affect *C. difficile* viability. **(A)** Killing of *C. difficile* R20291 growth (OD_600_) in BHI media supplemented with small molecules at mid log growth phase (Compounds 1 through 11). **(B)** Total C. *difficile* R20291 vegetative cells and spores, or spores alone 6 h post exposure to small molecules in **(A)** (Compounds 1, 2, and 3) at a concentration of 10 μg/ml when compared to solvent 0.25% DMSO (DMSO, positive control), or 2 μg/ml vancomycin (Vanco, negative control). Data presented represent mean ± SEM of triplicate experiments. Statistical significance between positive control (solvent) and treatment groups was determined by Student’s parametric *t*-test with Welch’s correction (^∗^*p* < 0.05, ^∗∗^*p* < 0.01).

Kill kinetics of *C. difficile* were evaluated by measuring the optical density (OD_600_) after the addition of 2-AI molecules to cells in mid log growth phase (**Figure 4A**). Supplementation of vancomycin (2 μg/ml) and solvent (0.25% DMSO) were used as controls. Compounds 1, 2, and 3 (10 μg/ml) altered growth, which was further confirmed by enumerating total colony forming units (CFUs) of vegetative cells and spores at the 6 h time point in **Figure 4B**. Addition of 2-AI molecules resulted in a significant log reduction in the total number of vegetative cells and spores for compound 1 (5.5 ± 0.57 log), compound 2 (4.0 ± 0.26 log), and compound 3 (3.6 ± 0.27 log) compared to the solvent control. However, no differences were seen in spores. The solvent DMSO did not affect *C. difficile* kill kinetics like the vancomycin control. 2-AI molecules compound 5, 6, 8, and 10, that did not inhibit growth and toxin activity were rejected at this point in the pipeline. Compounds 1, 2, 3, 4, 7, 9, and 11 were advanced to the next step in the pipeline.

### 2-Aminoimidazole Molecules Do Not Alter *C. difficile* R20291 Sporulation

**FIGURE 5:**
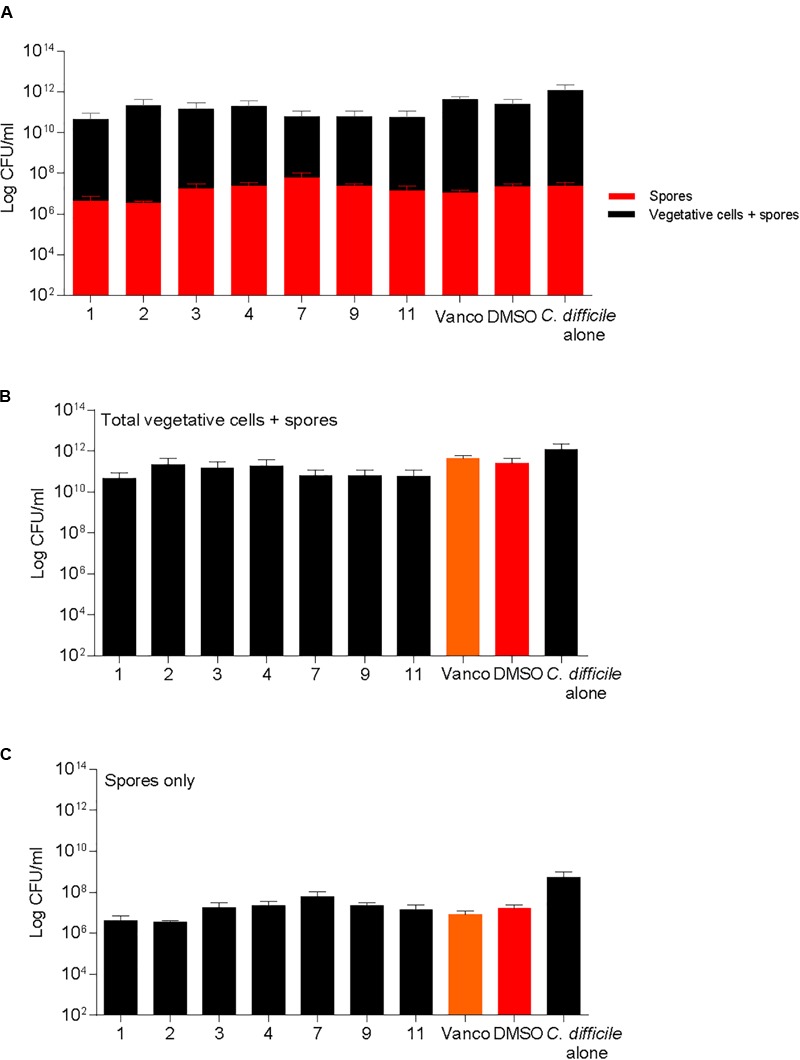
2-aminoimidazole molecules do not alter *C. difficile* sporulation. Sporulation of *C. difficile* on 70:30 agar plates after supplementation with 2-AI molecules (Compounds 1, 2, 3, 4, 7, 9, and 11) at a concentration of 10 μg/ml when compared to solvent 0.25% DMSO (DMSO, positive control), or 2 μg/ml vancomycin (Vanco, negative control) after 24 h. Data represents **(A)** Total vegetative cells and spores, and spores only, **(B)** Total vegetative cells and spores, **(C)** Spores only at 24 h post treatment.

Differences in sporulation were determined by inducing spore formation and quantification of heat resistant spores. Sporulation was unaffected when supplemented with DMSO or compounds 1, 2, 3, 4, 7, 9, and 11 (**Figure 5**). All molecules tested for sporulation were advanced to next step of screening in the pipeline. Spores were also enumerated in the kill assays described previously and no differences were noticed in the spores recovered in the BHI media both at 6 and 24 h post treatment (**Supplementary Figure S1**).

### 2-Aminoimidazole Molecules Affect Other *C. difficile* Strains Sparing Commensal Members of the Gut Microbiota

Compounds 1, 2, 3, 4, 7, 9, and 11 were screened for MICs against other *C. difficile* strains and a commensal microbiota library. Other *C. difficile* strains (CD196, M68, CF5, 630, BI9, and M120) were inhibited by compounds 1, 2, and 3 at a MIC of 2.5–5 μg/ml and 5–10 μg/ml, respectively (**Table 3**). Vancomycin was inhibitory to all strains at 0.31 μg/ml, except BI9, which was inhibited at 0.16 μg/ml. *C. difficile* strains were not susceptible to all other 2-AI molecules (compounds 4, 7, 9, and 11) at a concentration of 10 μg/ml.

**Table 3:** Minimum inhibitory concentration of 2-aminoimidazole molecules against other *C. difficile* strains compared to vancomycin.

*C. difficile* strain	MIC (μg/mL)			
	Compound 1	Compound 2	Compound 3	Vancomycin
CD196	5	5	5	0.31
M68	5	10	10	0.31
CF5	5	5	10	0.31
630	5	5	5	0.31
BI9	2.5–5	5	5	0.16
M120	5	10	10	0.31

*Minimum inhibitory concentration (MIC) was determined by broth microdilution as per modified CLSI guidelines for anaerobes. Data represent mean values from triplicate trials.*

Commensal microbes that are associated with a healthy gut microbiota and colonization resistance against *C. difficile*: *B. fragilis, B. thetaiotaomicron, C. scindens, C. hylemonae* were also susceptible to compound 1 at a MIC of 5–10 μg/ml (**Table 4**). In contrast, all other strains (*L. acidophilus, L. gasseri, E. coli, B. longum* subsp. *infantis*) remained resistant to compound 1 with a MIC greater than 10 μg/ml. Compound 2 was inhibitory to *B. thetaiotaomicron, C. scindens*, and *C. hylemonae* at 10 μg/ml. Interestingly, compound 3, which inhibited *C. difficile* growth, did not have any effect on the commensals at the tested concentration The commensal panel was resistant to compounds 4, 7, 9, and 11 at a concentration of 10 μg/ml, which also did not inhibit *C. difficile* strains.

**Table 4:** Minimum inhibitory concentration of 2-aminoimidazole molecules on commensal microbiota library.

Commensal strain	MIC (μg/mL)			
	Compound 1	Compound 2	Compound 3	Vancomycin
*B. fragilis*	10	>10	>10	2.5
*B. thetaiotaomicron*	5	10	>10	1.25
*L. acidophilus*	>10	>10	>10	0.31
*L. gasseri*	>10	>10	>10	0.16
*C. scindens*	5–10	10	>10	0.31
*C. hylemonae*	10	10	>10	1.25–0.31
*E. coli*	>10	>10	>10	>10
*B. longum* subsp. *infantis*	>10	>10	>10	0.63

*Minimum inhibitory concentration (MIC) was determined by broth microdilution as per modified CLSI guidelines for anaerobes. Data represent mean values from triplicate trials.*

## Discussion

In this study we developed and implemented a small molecule-screening pipeline to screen and select promising compounds that inhibited one or multiple steps in the *C. difficile* life cycle without altering the growth of a panel of gut commensals associated with colonization resistance. 2-AI molecules that have been successfully used to enhance antibiotic activity and mitigate virulence responses against other insidious pathogens were the first compounds screened through our pipeline. We evaluated eleven 2-AI molecules (compound 1 through 11) for their ability to alter *C. difficile* growth, toxin, and sporulation, while sparing other members of the gut microbiota. Compounds 1, 2, and 3 were microbicidal and were able to inhibit and kill *C. difficile* R20291 growth. The antimicrobial activity of compounds 1, 2, and 3 correlated with lower toxin activity. However, there was no difference in the number of spores recovered. Interestingly, compounds 4, 7, 9, and 11 were anti-virulent as they inhibited toxin activity without impacting the growth of *C. difficile* strains and commensals.

Minimum inhibitory concentrations of all molecules were first evaluated with *C. difficile* R20291, and then subsequently moved down the pipeline to evaluate how they affected growth kinetics, and virulence factors such as toxin and sporulation. Treatment with compound 1 (5.5 ± 0.57 log), compound 2 (4.0 ± 0.26 log), and compound 3 (3.6 ± 0.27 log) resulted in a higher log reduction of *C. difficile* vegetative cells and spores then the vancomycin control (2.7 ± 0.50). Based on MIC’s, vancomycin (0.15–0.31 μg/ml) was more potent against *C. difficile* R20291 compared to compounds 1, 2, and 3 (2.5–5 μg/ml). Similar sensitivity to vancomycin for *C. difficile* R20291 isolates has been reported (Barbut et al., 2007; Đapa et al., 2013; Brock, 2015). However, different antimicrobial sensitivity testing methods were used making it difficult to compare between studies. Since vancomycin is bacteriostatic to logarithmic phase cultures, it was not surprising that there was a lower log reduction at 2 μg/ml (Levett, 1991; Alam et al., 2015). Several antimicrobials with a range of modes of action are under clinical evaluation for CDI now (Kociolek and Gerding, 2016). Surotomycin is a novel lipopeptide that has antibacterial activity by disrupting the bacterial cell membrane (Mascio et al., 2012). It has potent activity against *C. difficile* and reduced activity against commensal bacteria (Citron et al., 2012). However, it was not associated with lower recurrence rates in phase III clinical trials (Boix et al., 2017). Cadazolid is another novel oxazolidinone compound which inhibits protein synthesis (Locher et al., 2014a). This compound reduces toxin production and sporulation *in vitro* in the absence of bacterial killing (Locher et al., 2014b). Ridinilazole a DNA synthesis inhibitor is a novel narrow spectrum antibiotic and has shown promising phase II results (Basseres et al., 2016; Steinebrunner et al., 2018). The mode of action for compounds 1, 2, and 3 screened in our study is unknown, and more studies are needed to explore bactericidal targets including cell wall biosynthesis, DNA replication, and protein synthesis.

Targeting virulence is a therapeutic approach that provides promising opportunities to inhibit pathogenesis *in vivo* without affecting bacterial growth (Cegelski et al., 2008). Mitigating virulence shifts the advantage to the host since the immune response remains unimpaired by the bacteria. Additionally, the gut microbiota that provide colonization resistance against *C. difficile* are unaltered, reducing recurrence. Common anti-toxin agents pursued as potential therapeutics for various infectious diseases include inhibitors of toxin transcription factors (Hung et al., 2005), toxin trafficking molecules (Saenz et al., 2007), and the use of toxin neutralizing antibodies (Arnon et al., 2006). Quorum sensing molecules (Hentzer et al., 2003) and bacterial two-component response systems that are central to bacterial virulence are often targeted for anti-virulence effect as well. In our study, compounds 4, 7, 9, and 11 did not affect growth, yet toxin activity decreased significantly compared to the solvent control. This is in line with the mechanism of action of 2-AI molecules that are able to target response regulator protein of bacterial TCS, thereby inhibiting virulence determinants such as antibiotic resistance, toxin secretion, and biofilm formation in other antibiotic resistant bacteria including *P. aeruginosa, A. baumannii*, and *S. aureus* (Rogers et al., 2010; Brackett et al., 2014; Draughn et al., 2017). In *C. difficile*, TCS is a part of the quorum sensing system called accessory gene regulator (agr) system that regulates toxin synthesis (Darkoh et al., 2015). The components of the agr system in strain R20291 includes *agrB1* and *agrD1* within the *agr1* loci, that are responsible for producing the quorum signaling autoinducer peptide, and *agrB2D2* and *agrC2A2* within *agr2* loci that are quorum signal-generation and response genes, respectively (Darkoh et al., 2016). *C. difficile* also has a Spo0A histidine kinase TCS system that is known to play a key role in both sporulation and toxin production (Underwood et al., 2009). However, the molecular mechanisms that lead to the control of toxin production by Spo0A are found to be strain dependent, and are not well characterized (Darkoh et al., 2015; Martin-Verstraete et al., 2016). Inhibition of any components in the accessory gene regulator pathway, and Spo0A histidine kinase TCS system could result in significant control of the toxin.

Targeting the toxin protein itself rather than bacterial growth to treat CDI is gaining momentum especially after *tcdA* and *tcdB* knockouts of toxigenic *C. difficile* proved to be avirulent in a hamster model (Kuehne et al., 2014). Both toxins are composed of four large domains: putative receptor binding domain, a transmembrane domain, a CPD, and a glucosyltransferase domain, whose conformational changes and the subsequent events leads to cytopathic and cytotoxic effect of the toxins (Pruitt and Lacy, 2012). These domains are potential drug targets for toxin inactivation. Bezlotoxumab an injectable human monoclonal antibody was FDA approved recently for the prevention of recurrent CDI. The antibodies bind to the receptor binding domain of toxin B when given systemically, thereby mitigating the *in vivo* effects of the toxin (Yang et al., 2015; Wilcox et al., 2017). A viable alternate strategy to target toxins is by using small molecules that could be delivered directly to the site of infection rather than systemic administration. Indeed, a promising bioactive compound, ebselen, which is currently under clinical investigation for unrelated indication was found to inhibit CPD activity *in vitro*. Ebselen was also validated in a mouse model to bind toxin B, and thereby prevent *C. difficile* induced clinical pathology (Bender et al., 2015). In another study using a chemical genetics strategy, several small molecules were screened to target potential domains and pathways. This study laid the foundation for identifying first-generation inhibitors of toxin B that mediate CDI (Tam et al., 2015). Antitoxin molecules represent a novel paradigm and could provide the industry with new opportunities in the treatment and management of CDI.

Since 2-AI molecules could potentially affect Spo0A histidine kinase TCS system that controls sporulation, we attempted to measure the inhibitory activity of 2-AI molecules on sporulation induction of mid log *C. difficile* cells. No differences were observed in the number of spores recovered with or without the addition of 2-AI molecules at a concentration of 10 μg/mL. Compounds 1, 2, and 3 were growth inhibitory at this concentration, however, it is crucial to evaluate if the 2-AI molecules induce stress on the cells resulting in increased spore formation. Fidaxomicin is the only drug currently available that inhibits sporulation when sub inhibitory concentrations are added to early stationary phase cells (Babakhani et al., 2012). Anti-sporulation properties would provide greater effectiveness to control transmission and reduce recurrences of CDI.

Since the gut microbiota plays a key role in providing colonization resistance against *C. difficile* (Theriot et al., 2014; Buffie et al., 2015), we tested the small molecules against eight different bacterial strains that are members of the healthy human gut microbiota, and six other *C. difficile* strains from distinct PCR ribotypes. We included members from four of the five dominant phyla of the gut microbiota including Firmicutes, Bacteroidetes, Actinobacteria, and Proteobacteria (Tremaroli and Backhed, 2012). Firmicutes make up 50–70% of the colonic bacterial community (Frank and Pace, 2008). Members of Firmicutes including *L. acidophilus, L. gasseri, C. scindens, C. hylemonae, C. heranonis* were added to the panel. *B. fragilis* and *B. sterocis* belonging to the phyla Bacteroidetes were added to the panel as they are designated as key stone species in the human gut microbiome (Fisher and Mehta, 2014). Another member of Bacteroidetes added was *B. thetaiotaomicron*. This commensal is found to antagonize intestinal pathogens through a range of mechanisms (de Sablet et al., 2009; Ferreira et al., 2011; Kamada et al., 2012). *B. infantis* belonging to the phyla Actinobacteria known to synthesize compounds necessary for functional maturation of enterocytes and host immunity, were also added to the panel (Round and Mazmanian, 2009; Guinane et al., 2011). Compounds 4, 7, 9, and 11 used at a concentration that inhibited *C. difficile* toxin activity had no effect on the commensal panel. Compounds 1 and 2 were microbicidal to *C. difficile*, but remained resistant to most of the commensal panel except for Bacteroides and the commensal *Clostridia*. Compound 3 had narrow spectrum activity against *C. difficile*, and did not affect growth of the commensal microbiota at a concentration of 10 μg/ml.

Screening novel small molecules against *C. difficile* rely on MIC assays or growth inhibition assays by measuring optical density in a plate reader. This is not always an accurate readout as exposure of *C. difficile* to stressors is able to increase sporulation (Wilcox and Fawley, 2000; Fawley et al., 2007). A drop in optical density overtime in a growth inhibition assay does not distinguish between vegetative cell lysis and spore formation. It is also important to evaluate viable counts of vegetative cells and spores to confirm true growth inhibition. In this study, growth was evaluated in multiple assays including a growth kinetics inhibition assay (microbroth dilution technique and OD_600_ measurement on cells in early log phase), and a kill kinetics assay (OD_600_ measurement and bacterial enumeration of cells in mid log phase). Another strength of our pipeline is that it takes into consideration other *C. difficile* strains from distinct ribotypes to ensure there are no differences in susceptibility across strains. Additionally, understanding how these compounds affect other gut commensal bacteria is important for the restoration of colonization resistance *in vivo.* The pipeline not only allows for quick screening of antimicrobials, but also for anti-virulence agents. The test molecule concentrations selected for screening can be modified based on each molecule.

There are many strengths to using this small-molecule screening pipeline, however, there are some limitations. We did not evaluate the first stage of the *C. difficile* life cycle, spore germination. However, addition of this assay to the pipeline in the future could be valuable. Although the Vero cell cytotoxicity assay we use in this study is the gold standard for evaluating toxin activity it is semi-quantitative, and other assays such as qRT-PCR and immunoblotting are more quantitative. Another limitation of our toxin assay is that the BHI media used for culturing was supplemented with cysteine, which can reduce toxin expression (Karlsson et al., 2000; Dubois et al., 2016). However, controls using the same media were used for comparison which ensures equal impact across all treatments. The sporulation assay also has limitations as it evaluates sporulation induction when test molecules are added ≥MICs and incubated for 24 h. Therefore, the results of the sporulation assay were not used as a criterion to move the test molecules to the next level of screening. Further testing evaluating sporulation inhibition could be done by adding sub-inhibitory concentrations of test molecules to *C. difficile* cultures and allowing an extended period of incubation before spore enumeration.

Finally, future studies are needed to characterize the anti-toxin activity and understand the mode of action for these 2-AI compounds. The next step after completing the pipeline is to test the therapeutic properties of 2-AI molecules in a mouse model. Etiology of CDI is complex and a combined approach of drugs inhibiting different stages of *C. difficile* life cycle are advantageous for the treatment and management of CDI.

## Author Contributions

RT, DZ, RD, and CT conceived and designed the experiments. RT performed the experiments. RT and CT performed the analysis. RT, DZ, RD, and CT wrote and edited the manuscript.

## Conflict of Interest Statement

CT is a scientific advisor to Locus Biosciences, a company engaged in the development of antimicrobial technologies. DZ and RD are employees of Agile Sciences, Inc., a company engaged in the development of antimicrobial technologies. The other author declares that the research was conducted in the absence of any commercial or financial relationships that could be construed as a potential conflict of interest.

## Supplementary Material

FIGURE S1 2-aminoimidazole molecules do not alter *C. difficile* spores recovered in BHI media at 6 and 24 h post treatment. **(A)** Total C. *difficile* R20291 vegetative cells and spores, **(B)** total vegetative cells and spores, **(C)** spores only at 6 h and **(D)** total C. *difficile* R20291 vegetative cells and spores, **(E)** total vegetative cells and spores, **(F)** spores only at 24 h for Compounds 1, 2, and 3 at a concentration of 10 μg/ml when compared to solvent 0.25% DMSO (DMSO, positive control), or 2 μg/ml vancomycin (Vanco, negative control). Data presented represent mean ± SEM of triplicate experiments. Statistical significance between positive control (solvent) and treatment groups was determined by Student’s parametric *t*-test with Welch’s correction (^∗^*p* < 0.05, ^∗∗^*p* < 0.01).

## Abbreviations

2-AI: 2-aminoimidazole
CDC: The Centers for Disease Control and Prevention
CDI: *C. difficile* infection
CPD: cysteine protease domain
MIC: minimum inhibitory concentration
