# Supplementary figures and images for: A Small Molecule-Screening Pipeline to Evaluate the Therapeutic Potential of 2-Aminoimidazole Molecules Against Clostridium difficile
Source: Front Microbiol. 2018 Jun 6;9:1206. doi: 10.3389/fmicb.2018.01206 (PMC5997789; doi:10.3389/fmicb.2018.01206)

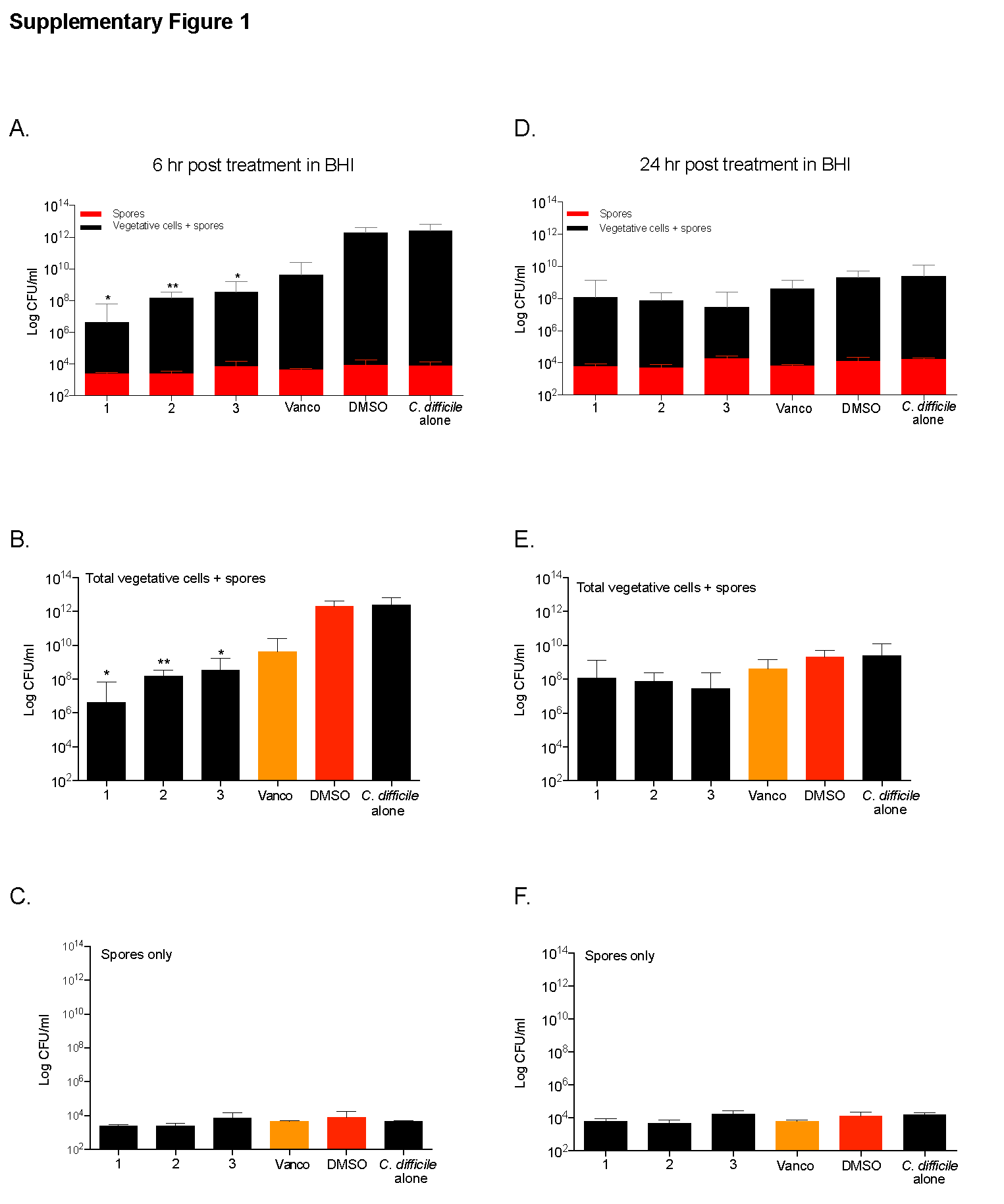

Supplement: FIGURE S1 — 2-aminoimidazole molecules do not alter C. difficile spores recovered in BHI media at 6 and 24 h post treatment. (A) Total C. difficile R20291 vegetative cells and spores, (B) total vegetative cells and spores, (C) spores only at 6 h and (D) total C. difficile R20291 vegetative cells and spores, (E) total vegetative cells and spores, (F) spores only at 24 h for Compounds 1, 2, and 3 at a concentration of 10 μg/ml when compared to solvent 0.25% DMSO (DMSO, positive control), or 2 μg/ml vancomycin (Vanco, negative control). Data presented represent mean ± SEM of triplicate experiments. Statistical significance between positive control (solvent) and treatment groups was determined by Student’s parametric t-test with Welch’s correction (∗p < 0.05, ∗∗p < 0.01). [file Image_1.TIFF]
